# Psychometric reliability of patient-reported visual analogue scales in subthalamic nucleus deep brain stimulation programming for Parkinson’s disease

**DOI:** 10.1093/braincomms/fcag100

**Published:** 2026-03-19

**Authors:** Johannes Off, Maximilian Scherer, Sophia Peschke, Angelina Kirschner, Weidong Zhang, Juhi Shaik, Jing Dong, Jan-Hinnerk Mehrkens, Elisabeth Kaufmann, Thomas Koeglsperger

**Affiliations:** Department of Neurology, Ludwig Maximilian University, 81377 Munich, Germany; Department of Neurology, Ludwig Maximilian University, 81377 Munich, Germany; Department of Neurology, Ludwig Maximilian University, 81377 Munich, Germany; Department of Neurology, Ludwig Maximilian University, 81377 Munich, Germany; Department of Neurology, Ludwig Maximilian University, 81377 Munich, Germany; Department of Neurology, Ludwig Maximilian University, 81377 Munich, Germany; Department of Neurology, Ludwig Maximilian University, 81377 Munich, Germany; Department of Neurosurgery, Ludwig Maximilian University, 81377 Munich, Germany; Department of Neurology, Ludwig Maximilian University, 81377 Munich, Germany; Department of Neurology, Ludwig Maximilian University, 81377 Munich, Germany; Department of Translational Brain Research, German Centre for Neurodegenerative Diseases (DZNE), 81377 Munich, Germany

**Keywords:** visual analogue scale (VAS), Parkinson’s disease (PD), deep brain stimulation (DBS)

## Abstract

Subthalamic nucleus deep brain stimulation is an established therapy for Parkinson’s disease, yet its programming relies heavily on subjective patient feedback. Visual analogue scales have been proposed to structure patient-reported outcome measures during programming, but their psychometric reliability has not been systematically evaluated. In this study, fifteen patients with bilateral subthalamic nucleus deep brain stimulation completed four structured experiments to assess the reliability of visual analogue scales: test–retest consistency, the effect of stimulation duration (15, 60, 120 s), the impact of unilateral deep brain stimulation withdrawal intervals (0, 10, 30 min), and contralateral stimulation ON versus OFF. Across all experiments, patients provided over 3000 visual analogue scale ratings, which were analyzed using correlation, regression, and Bland–Altman methods, with subgroup analyses examining motor phenotype, cognition, and disease burden. Visual analogue scale ratings demonstrated strong test–retest reliability (*r = *0.70, *R*^2^ = 0.53), with 83% of repeated scores within ±2 points. Reliability was lower in patients with tremor-onset compared to non-tremor onset (*P* = 0.04) but was unaffected by cognitive status or quality of life. Stimulation duration influenced absolute scores, with 15 s ratings systematically lower than 60–120 s (*P* < 0.001), though relative scaling was preserved. Deep brain stimulation withdrawal intervals did not affect group means but increased trial-level variability, while contralateral stimulation ON versus OFF showed modest correspondence (*r = *0.31, *R*^2^ = 0.13), suggesting hemispheric interactions in subjective perception. These findings indicate that visual analogue scale ratings provide reproducible and quantifiable feedback during subthalamic nucleus deep brain stimulation programming. Exploratory analyses suggest that reliability may vary with motor phenotype, stimulation duration, and bilateral context. Incorporating structured visual analogue scale feedback could enhance programming workflows, support remote care models, and inform future multimodal closed-loop deep brain stimulation strategies.

## Teaser

Off *et al*. show that visual analogue scale ratings provide reliable feedback during subthalamic nucleus deep brain stimulation programming in Parkinson’s disease. Reliability varies with motor phenotype, stimulation duration, and bilateral stimulation. Structured visual analogue scale feedback supports clinical programming, remote monitoring, and development of adaptive deep brain stimulation systems.

## Graphical Abstract

**Graphical Abstract:**
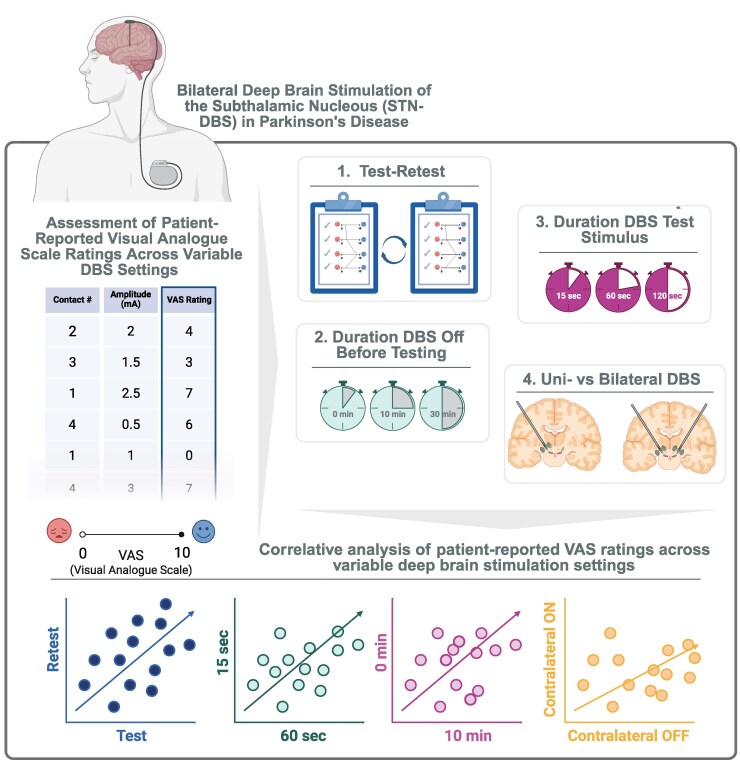

## Introduction

Deep brain stimulation (DBS) targeting the subthalamic nucleus (STN) is a well-established and effective treatment for patients with advanced Parkinson’s disease (PD), offering substantial and sustained improvements in motor function, medication burden, and quality of life.^1,2^ Following surgical implantation, clinical benefit depends critically on postoperative programming—a process that involves systematic testing of stimulation parameters to identify an optimal therapeutic window. This involves balancing motor symptom control with minimization of stimulation-induced side effects, such as dysarthria, paraesthesia, or mood changes. Despite increasing efforts to standardize programming protocols, the process remains highly individualized and inherently subjective, relying heavily on patient feedback and clinician interpretation.^3,4^

Visual analogue scales (VAS) are widely used psychometric instruments designed to capture subjective experiences across a continuous spectrum. VAS provides a simple measure of symptom perception by asking patients to rate their perceived benefit or side effects on a linear scale.^5^ Their ease of use, minimal cognitive demand, and broad applicability have made them a standard tool in clinical research and practice across domains including pain,^6^ anxiety,^7^ and sensory processing.^8^ Recently, we proposed VAS as a method to systematically structure patient-reported outcome measures (PROMs) during DBS programming, offering a potential means of quantifying subjective feedback to support data-driven programming decisions.^9,10^

Some DBS effects may take hours to days to fully manifest, whereas others diminish over time due to habituation.^3,11^ Additionally, interactions between bilateral stimulation settings, residual effects from prior configurations, and patient expectations may introduce contextual variability in reported ratings. While VAS-based approaches have shown promise in structured programming paradigms, the extent to which these ratings are consistent over time, and across different stimulation conditions, and experimental contexts has never been examined.

To our knowledge, the psychometric reliability of VAS ratings in the context of STN-DBS programming for PD has not been systematically assessed. Understanding the stability of these measures is a critical step towards their broader integration into clinical workflows and research protocols. This study aims to determine whether patient VAS ratings remain stable across experimental conditions relevant to STN-DBS programming, including variations in electrode configuration, stimulation duration, washout period, and repeated testing.

## Materials & methods

### Study Participants

The study was approved by the local ethics committee of Ludwig Maximilian University of Munich, Germany (#18-0809), and all patients gave written informed consent. Participants were recruited between June 2024 and March 2025 during routine outpatient visits. Inclusion criteria were: PD diagnosed per MDS criteria, bilateral STN-DBS implanted ≥12 months, and stable stimulation settings for ≥3 months. Exclusion criteria were conditions limiting communication, consent, or compliance, and manifest dementia per ICD-10. Seventeen patients initially consented to participate in the study. Two participants discontinued participation before completing Experiment I, resulting in a final sample of fifteen patients for the primary test–retest reliability analysis. Due to participant burden and scheduling constraints, not all participants completed all experimental paradigms: thirteen participants completed Experiments II and III, and nine participants completed Experiment IV. Analyses for each experiment were therefore conducted using all available data, and no imputation for missing data was performed.

### Study Visit & Experimental Procedures

**Figure 1:**
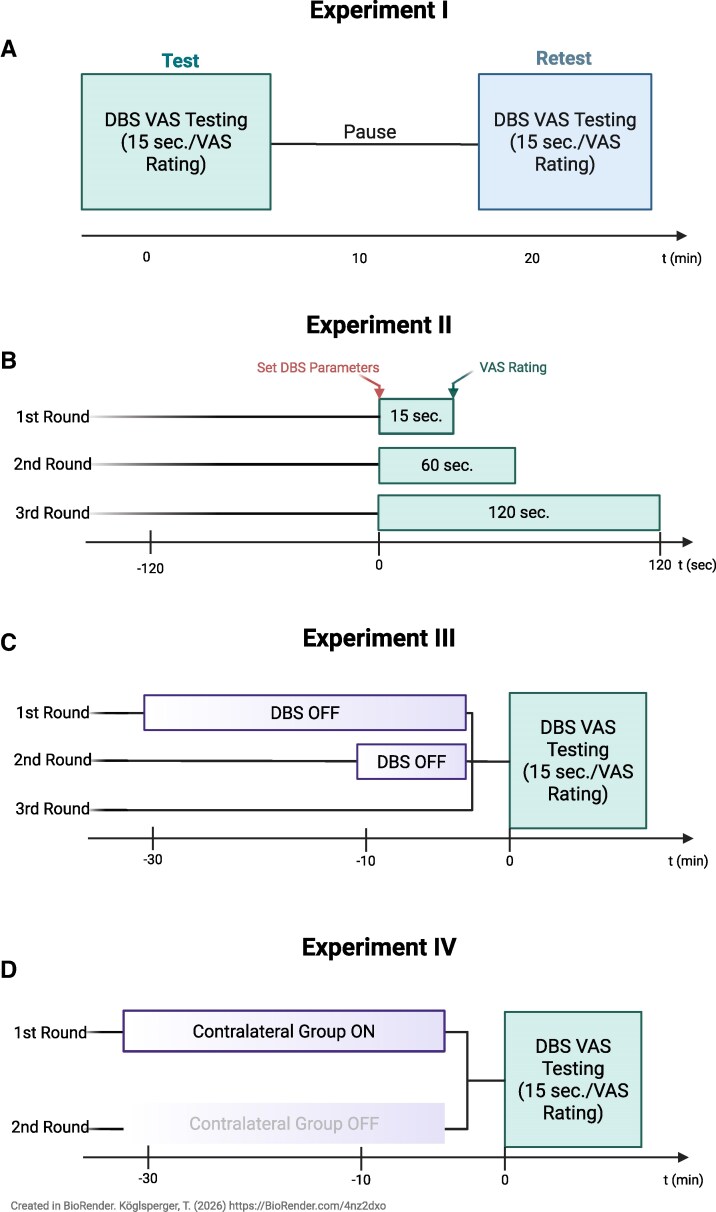
**Experimental workflow.** (A-D) Schematic illustrating the workflow for the experiments I-IV.

At the start of each visit, chronic stimulation parameters were recorded, and clinical assessments were performed in the Med On/Stim On condition, including Movement Disorder Society–Unified Parkinson’s Disease Rating Scale (MDS-UPDRS), Montreal Cognitive Assessment (MoCA), and Parkinson’s Disease Questionnaire-39 (PDQ-39). Participants completed four structured experiments (I–IV) across separate sessions (Fig. 1), with 5–10 min rest periods between experiments to minimize fatigue and carry-over effects. Clinical stimulation settings were restored after each experiment.

#### Experiment I—Test–Retest Reliability

For each hemisphere, we generated two spreadsheets, each containing the same 18 combinations of stimulation contact and amplitude (herein termed programmes) (8 random contacts at 1 mA, 8 random contacts at 3 mA, and 2 no-stimulation controls, all at f = 130 Hz and pw = 60 μs) (Supplementary Table 1), with separate versions for non-directional and directional leads. The second spreadsheet randomized the programme order. During testing, stimulation programmes were applied sequentially according to the first spreadsheet. Each programme was switched on for 15 s, after which the patient rated their percept on a VAS as previously described.^9,10^ In brief, patients were asked to rate the overall quality of the DBS effect following each stimulation adjustment on a scale from 0 to 10 (0 = ‘very bad,’ 10 = ‘very good’). No further instructions were provided, and interpretation of intermediate values was left to the patient’s discretion. Participants were blinded to the specific stimulation parameters. In cases where stimulation induced intolerable side effects, the corresponding contact was excluded from further testing at higher amplitudes. A 15 s washout period followed before the next program was activated. Once all 18 programs were completed, the retest session for the same hemisphere was conducted after a 5-minute pause, using the identical programs presented in a randomized order, with randomization pre-specified and performed using a web-based tool. (Supplementary Table 1, Fig. 1A). Only after both test and retest were completed in the first hemisphere did the procedure proceed to the contralateral side, which remained switched on throughout testing. The same two-session protocol was repeated contralaterally, resulting in 36 ratings per hemisphere (18 test + 18 retest) and 72 VAS scores per participant.

#### Experiment II—Effect of the duration of the test stimulus

For each hemisphere, 18 stimulation programs were defined as described above (8 contacts at 1 mA, 8 contacts at 3 mA, and 2 no-stimulation controls; (Supplementary Table 2), with the contralateral side switched on throughout testing. Each hemisphere was tested three times using the same stimulation programs but varying the duration of the test stimulus before the VAS rating (Supplementary Table 2; Fig. 1B). In the first pass, each program was activated for 15 s, after which the patient provided a VAS rating. All 18 programs were presented sequentially. In the second pass, the same programs were repeated in a new randomized order, with stimulation maintained for 60 s before each VAS rating. Finally, the programs were presented a third time in randomized order, with stimulation lasting 120 s before the patient rated each program. Ratings for the 15-second condition were identical to those obtained in Experiment I. Across both hemispheres and all three duration conditions; each participant provided a total of 108 VAS ratings (3 × 36).

#### Experiment III—Stimulation Withdrawal Before Testing

This experiment investigated how the duration of DBS withdrawal prior to testing affected patient VAS ratings. For each participant, the DBS device on the hemisphere being tested was turned off unilaterally for 0, 10, or 30 min. Following each period with stimulation off, participants rated all 18 individual contact settings presented in a new randomized order, with randomization pre-specified and performed using a web-based tool (Supplementary Table 3; Fig. 1C). Ratings for the 0-minute condition were identical to those obtained in Experiment I. Testing was conducted in three consecutive rounds. In the first round, the side to be tested had the DBS switched off for 30 min before the 18 programs were sequentially activated, with patients given 15 s to provide a VAS rating for each program. In the second round, the same programs were presented after a 10-minute stimulation-off period, and in the third round, the programs were presented without any prior stimulation withdrawal. In each round, the programs were presented in a new randomized order. Across all three rounds and both hemispheres, each participant provided a total of 108 VAS ratings (3 × 36).

#### Experiment IV—contralateral off comparison

This experiment replicated the procedure of Experiment I but specifically assessed the impact of unilateral versus bilateral stimulation. For each participant, the contralateral lead was turned off while the ipsilateral side was tested. Participants rated all 36 individual contact settings (18 per hemisphere) with the contralateral hemisphere unstimulated. The order of contacts was randomized using a new sequence unique to this experiment with randomization pre-specified and performed using a web-based tool. (Supplementary Table 4; Fig. 1D). Ratings for the contralateral On condition were reused from Experiment I. Across both hemispheres, each participant provided a total of 72 VAS ratings (2 × 36).

### Statistical analysis

All statistical analyses were performed using GraphPad Prism (version 10.4.1).

#### Experiment I

Reliability and reproducibility of VAS ratings were assessed via paired comparisons across repeated/matched conditions. Spearman’s rank correlation (r_s_) quantified monotonic relationships, accommodating non-normality, interpreted as |r| > 0.7 (strong), 0.5–0.7 (moderate), < 0.5 (weak). Two-tailed *P*-values and 95% CIs were reported. Linear relationships were evaluated using simple linear regression (slope, intercept, 95% CIs, *R*^2^), and slopes across stimulation durations were compared via regression with an interaction term (VAS score ∼ predictor × duration) using an F-test. Agreement was assessed with Bland–Altman analysis (bias ± 1.96 × SD). Within-patient variability was quantified by modelling test–retest differences with a Gaussian distribution; proportions within ±1 and ±2 VAS points were calculated, with differences > ±2 indicating perceptual inconsistency. Subgroup analyses examined effects of motor symptom at onset (tremor versus non-tremor), tremor severity (MDS-UPDRS III), cognitive status (MoCA <26 versus ≥ 26), and quality of life (PDQ-39 median split), repeating correlation and regression analyses and comparing slopes across durations (F-test). All tests were two-tailed, with *P* < 0.05 for significance.

#### Experiment II

Effects of stimulus duration (15, 60, 120 s) were evaluated. Pairwise correlations assessed consistency across time points. Spearman correlations and linear regression (baseline VAS → outcome, stratified by duration) examined predictive relationships, with slopes compared across durations (*F*-test). Absolute VAS differences across durations were analyzed via Friedman test with Dunn’s post hoc comparisons.

#### Experiment III

Effects of DBS-off time (0, 10, 30 min) were evaluated. Pairwise correlations assessed consistency, and Spearman correlations and linear regression (baseline VAS → outcome, stratified by Off duration) examined predictive relationships. Slopes were compared across durations (F-test), and group-level differences were tested via Friedman test with post hoc correction. To compare the median difference between groups, 5000 bootstrap samples were taken as described (www.estimationstats.com).^12^

#### Experiment IV

Influence of contralateral stimulation was assessed by comparing unilateral (contralateral Off) versus bilateral (contralateral On) VAS ratings. Associations were evaluated via Spearman correlation and linear regression (slope, intercept, *R*^2^). To compare the median difference between groups, 5000 bootstrap samples were taken as described (www.estimationstats.com).^12^

## Results

### Participant Characteristics

Seventeen patients initially consented to participate in the study. Two participants discontinued early, resulting in a final sample of fifteen patients who completed Experiment I. Of these, thirteen completed Experiments II and III, and nine completed Experiment IV (Table 1). 7 participants were female, with a mean age of 67,1± 5,2 years. 10 participants were male, with a mean age of 68,0 ± 5,6 years. Overall, the mean age was 67,6 ± 5,4 (mean ± SD) years across all 17 participants. The average disease duration was 16,8 ± 3,6 years, and the average duration of DBS treatment was 5,6 ± 2,8 years. MDS-UPDRS Part III scores at the study visit were 22,1 ± 11,8 in the medication On/stimulation On condition. Three patients were implanted with non-directional electrodes, while fourteen had segmented (directional) leads.

**Table 1:** Demographic and clinical characteristics of the study cohort

	N	female	Mean Age (yrs.)	Mean disease duration	Mean duration of DBS treatment	Directional leads	UPDRS-Score (I-IV)	UPDRS-Score I	UPDRS-Score II	UPDRS-Score III	UPDRS-Score IV	PDQ-39 SI	MoCA
**Experiment 1**	15	0,33 (5/15)	68,2 ± 5,7	16,5 ± 3,7	5,7 ± 3,0	0,80 (12/15)	49,3 ± 18,2	11,0 ± 4,2	14,7 ± 7,7	22,1 ± 11,6	2,5 ± 3,4	28,0 ± 12,4	26,1 ± 2,9
**Experiment 2**	13	0,23 (3/13)	67,7 ± 6,0	16,9 ± 3,8	5,9 ± 3,1	0,77 (10/13)	48,8 ± 18,0	10,8 ± 4,2	15,1 ± 7,8	21,7 ± 11,7	2,3 ± 3,4	27,3 ± 12,6	26,1 ± 3,1
**Experiment 3**	13	0,23 (3/13)	67,7 ± 6,0	16,9 ± 3,8	5,9 ± 3,1	0,77 (10/13)	48,8 ± 18,0	10,8 ± 4,2	15,1 ± 7,8	21,7 ± 11,7	2,3 ± 3,4	27,3 ± 12,6	26,1 ± 3,1
**Experiment 4**	9	0,22 (2/9)	68,8 ± 6,1	15,4 ± 3,1	5,1 ± 3,0	0,89 (8/9)	50,2 ± 20,8	11,7 ± 2,9	15,4 ± 7,6	21,7 ± 12,4	1,4 ± 2,3	27,7 ± 11,2	25,6 ± 3,5

### Test–retest reliability

**Figure 2:**
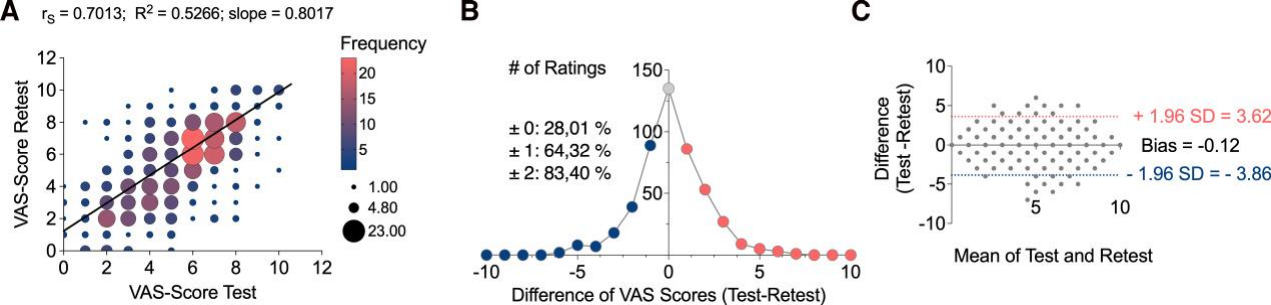
**Contextual modulation of VAS rating reliability during DBS programming.** (**A**) Bubble plot showing the relationship between test and retest VAS scores across 15 patients (n = 482 paired observations). Bubble size reflects overlapping data points. Spearman correlation demonstrated strong test–retest reliability (*r*_S_ = 0.7013, *P* < 0.0001). (**B**) Gaussian distribution of intra-individual test–retest differences. Most values fell within ±1 VAS point (64.3%) or ±2 points (83.4%), indicating limited intra-individual variability. Mean difference = −0.12, SD = 1.99. (**C**) Bland–Altman plot illustrating agreement between test and retest VAS scores. The mean bias was −0.12, with 95% limits of agreement from −3.87 to +3.84, calculated as mean ± 1.96 × SD. Each point represents the difference of one paired observation (experimental unit = patient).

To assess test–retest reliability of the VAS, correlation and regression analyses were performed on paired scores from n = 482 individual VAS ratings (Supplementary Table 1). Spearman's rank correlation revealed a strong and statistically significant association between VAS test and retest scores (r_S_ = 0.7013, 95% CI: 0.6513–0.7452; *P* < 0.0001), indicating strong reliability. Simple linear regression further confirmed a strong linear relationship between test and retest scores (*R*^2^ = 0.5266; slope = 0.801; F(1480) = 533.8; *P* < 0.0001), suggesting that approximately 53% of the variance in retest scores was explained by initial test scores (Fig. 2A).

To further evaluate within patient variability, test–retest difference scores were modelled using a Gaussian distribution. Most values (64.32%) fell within ±1 VAS point, and 83.40% were within ±2 points reinforcing the overall reliability of patient-reported VAS ratings across repeated assessments (Fig. 2B).

To complement the correlation and regression findings, a Bland–Altman analysis was performed to assess agreement between individual test and retest scores (Fig. 2C). The mean difference (bias) was −0.12, with 95% limits of agreement ranging from −3.87 to +3.84. Approximately 95% of paired observations fell within the limits of agreement, as expected under normal assumptions, and no trend toward increasing or decreasing disagreement across the VAS scale was observed, suggesting no proportional bias.

### Subgroup analyses

**Figure 3:**
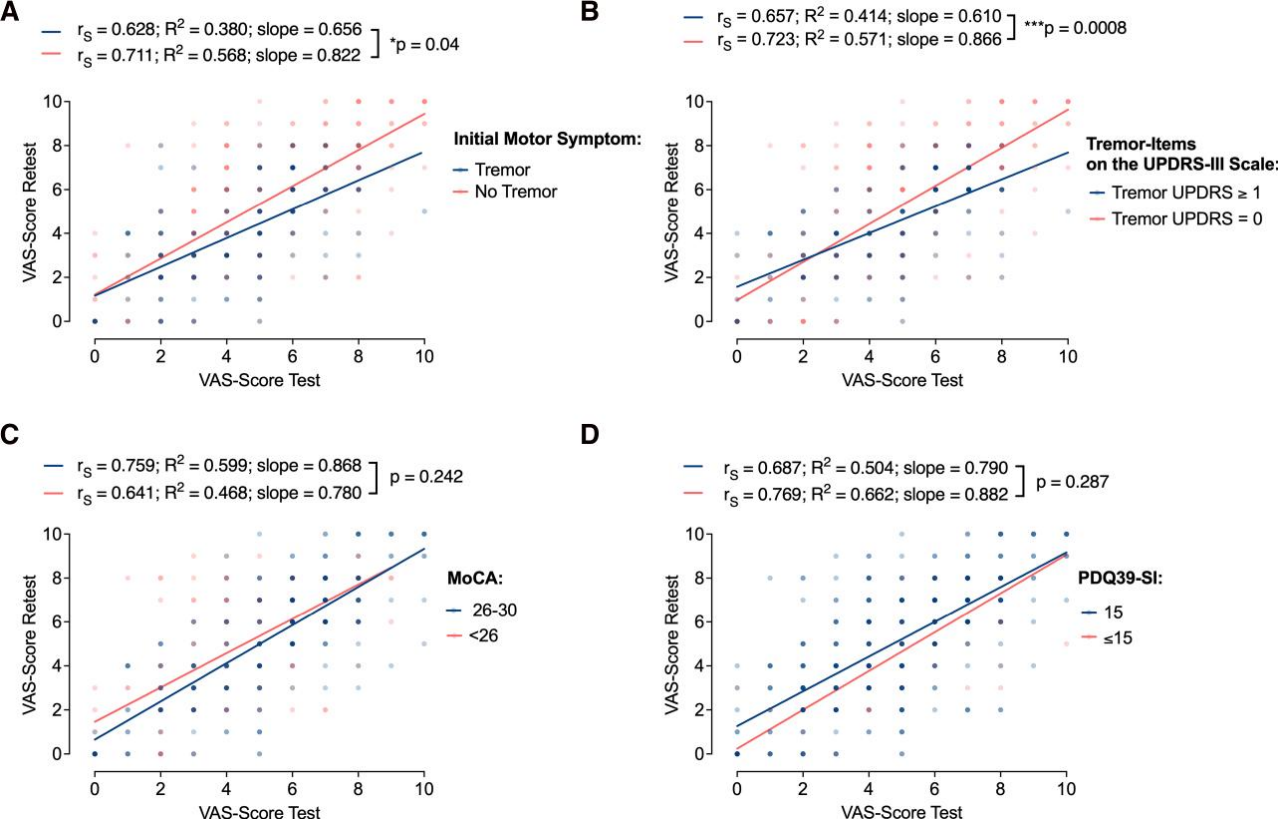
**Subgroup differences in VAS test–retest reliability.** Scatter plots illustrate the relationship between test and retest VAS scores across post hoc subgroups. Linear regression fits (solid lines) and Spearman rank correlations are shown for each group. Experimental unit = patient; n indicates number of patients per subgroup. (**A**) Stratified by self-reported motor symptom onset: tremor-onset (n = 4) versus non-tremor onset (n = 11). Regression slopes differed significantly (F(1479) = 4.226, *P* = 0.0404). Spearman correlations: *r*_S_ = 0.6287 (tremor-onset), *r*_S_ = 0.7119 (non-tremor). (**B**) Based on MDS-UPDRS Part III tremor score: tremor-positive (n = 5) versus tremor-negative (n = 10). Significant slope difference: F(1478) = 11.30, *P* = 0.0008. Spearman correlations: *r*_S_ = 0.6572 (tremor-positive), *r*_S_ = 0.7233 (tremor-negative). (**C**) Stratified by MoCA cognitive status: MoCA <26 (n = 5) versus MoCA ≥26 (n = 8). Slope comparison not significant (F(1432) = 1.373, *P* = 0.2420). Spearman correlations: *r*_S_ = 0.6412 (MoCA <26), *r*_S_ = 0.7594 (MoCA ≥26). (**D**) Grouped by PDQ-39 disease burden (median split, cut-off = 15): high-burden (n = 11) versus low-burden (n = 3). No significant slope difference (F(1478) = 1.135, *P* = 0.2873). Single data points are shown for the low-burden group (n < 10). Spearman correlations: *r*_S_ = 0.6878 (high-burden), *r*_S_ = 0.7693 (low-burden).

To assess the consistency of VAS test–retest reliability within post hoc defined subgroups, four analyses were conducted. First, participants were stratified based on self-reported motor symptoms at disease onset. Among those who reported tremor onset (*n *=4), the regression slope was 0.6563 (*R*^2^ = 0.3808), indicating a moderately strong linear relationship between test and retest VAS scores (Fig. 3A). In contrast, participants with non-tremor onset (*n *=11) exhibited a steeper slope of 0.8224 and higher *R*^2^ of 0.5681, suggesting greater consistency. A formal comparison of regression slopes revealed a statistically significant difference (F(1,  479) = 4.226, *P* = 0.0404), implying that initial motor phenotype may influence the reliability of subjective ratings. This pattern was corroborated by Spearman correlations, with non-tremor participants showing higher reliability (r_S_ = 0.7119, 95% CI: 0.6533–0.7621, *P* < 0.0001) than those with tremor onset (r_S_ = 0.6287, 95% CI: 0.5148–0.7207, *P* < 0.0001).

A complementary analysis based on MDS-UPDRS Part III tremor items classified patients as tremor-positive (score ≥1, *n *=5) or tremor-negative (score = 0, *n *=10) at the time of the study visit (Med On/Stim On). The tremor-positive group showed a slope of 0.6108 (*R*^2^ = 0.4149), while the tremor-negative group had a steeper slope of 0.8660 and higher *R*^2^ = 0.5714 (Fig. 3B). The group difference in slope was statistically significant (F(1,  478) = 11.30, *P* = 0.0008), suggesting that the absence of tremor doesn’t compromise consistency in perceptual scaling but improves its reliability. Spearman correlations aligned with this finding: r_S_ = 0.6572 (95% CI: 0.5573–0.7384, *P* < 0.0001) for tremor-positive, and *r = *0.7233 (95% CI: 0.6645–0.7732, *P* < 0.0001) for tremor-negative participants.

Stratifying participants by MoCA score (cut-off <26 for mild cognitive impairment),^13^ those with lower scores (*n *=5) showed a regression slope of 0.7805 (*R*^2^ = 0.4680), compared to a steeper slope of 0.8681 (*R*^2^ = 0.5997) in the cognitively preserved group (MoCA 26–30, *n *=8) (Fig. 3C). However, this difference was not statistically significant (F(1,  432) = 1.373, *P* = 0.2420). Spearman analyses confirmed reliable test–retest performance in both groups: r_S_ = 0.6412 (95% CI: 0.5431–0.7220, *P* < 0.0001) for MoCA <26, and r_S_ = 0.7594 (95% CI: 0.7003–0.8081, *P* < 0.0001) for MoCA ≥26. Two participants with missing MoCA data were excluded.

Using the PDQ-39 summary index, a median split (cut-off = 15) categorized patients into higher (*n *=11) and lower disease burden groups (*n *=3). The high-burden group showed a slope of 0.7901 (*R*^2^ = 0.5045), while the low-burden group demonstrated a slightly steeper slope of 0.8825 (*R*^2^ = 0.6625) (Fig. 3D). This difference was not statistically significant (F(1,  478) = 1.135, *P* = 0.2873). Both subgroups exhibited moderate to high test–retest reliability in Spearman analyses: r_S_ = 0.6878 (95% CI: 0.6286–0.7391, *P* < 0.0001) for the high-burden group and r_S_ = 0.7693 (95% CI: 0.6760–0.8383, *P* < 0.0001) for the low-burden group. One participant was excluded due to missing PDQ-39 data.

To determine whether disease severity influenced the reliability of subjective perceptual ratings, both correlation and linear regression analyses were conducted using scores from UPDRS Parts I–IV. Test–retest slope values served as the measure of reliability. Correlation analyses revealed no significant associations between reliability slopes and UPDRS subscores. Correlation coefficients were low and non-significant for all domains: UPDRS I (*r*_S_ = −0.06, *P* = 0.84), UPDRS II (*r*_S_ = 0.10, *P* = 0.74), UPDRS III (*r*_S_ = −0.20, *P* = 0.48), and UPDRS IV (*r*_S_ = −0.05, *P* = 0.87), indicating a lack of monotonic relationships between disease severity and perceptual reliability (Supplementary Fig. 1A-D). In parallel, simple linear regression analyses confirmed these findings. Regression slopes did not significantly differ from zero for any UPDRS subscore: UPDRS I (slope = −0.85, 95% CI: −9.55 to 7.85, *P* = 0.8350, *R*^2^ = 0.0038), UPDRS II (slope = 3.53, 95% CI: −14.13 to 21.19, *P* = 0.6710, *R*^2^ = 0.0156), UPDRS III (slope = −5.31, 95% CI: −30.96 to 20.34, *P* = 0.6621, *R*^2^ = 0.0152), and UPDRS IV (slope = −0.51, 95% CI: −8.02 to 7.00, *P* = 0.8852, *R*^2^ = 0.0017) (Supplementary Fig. 1E-F).

### Effect of Stimulation Duration on VAS Ratings

**Figure 4:**
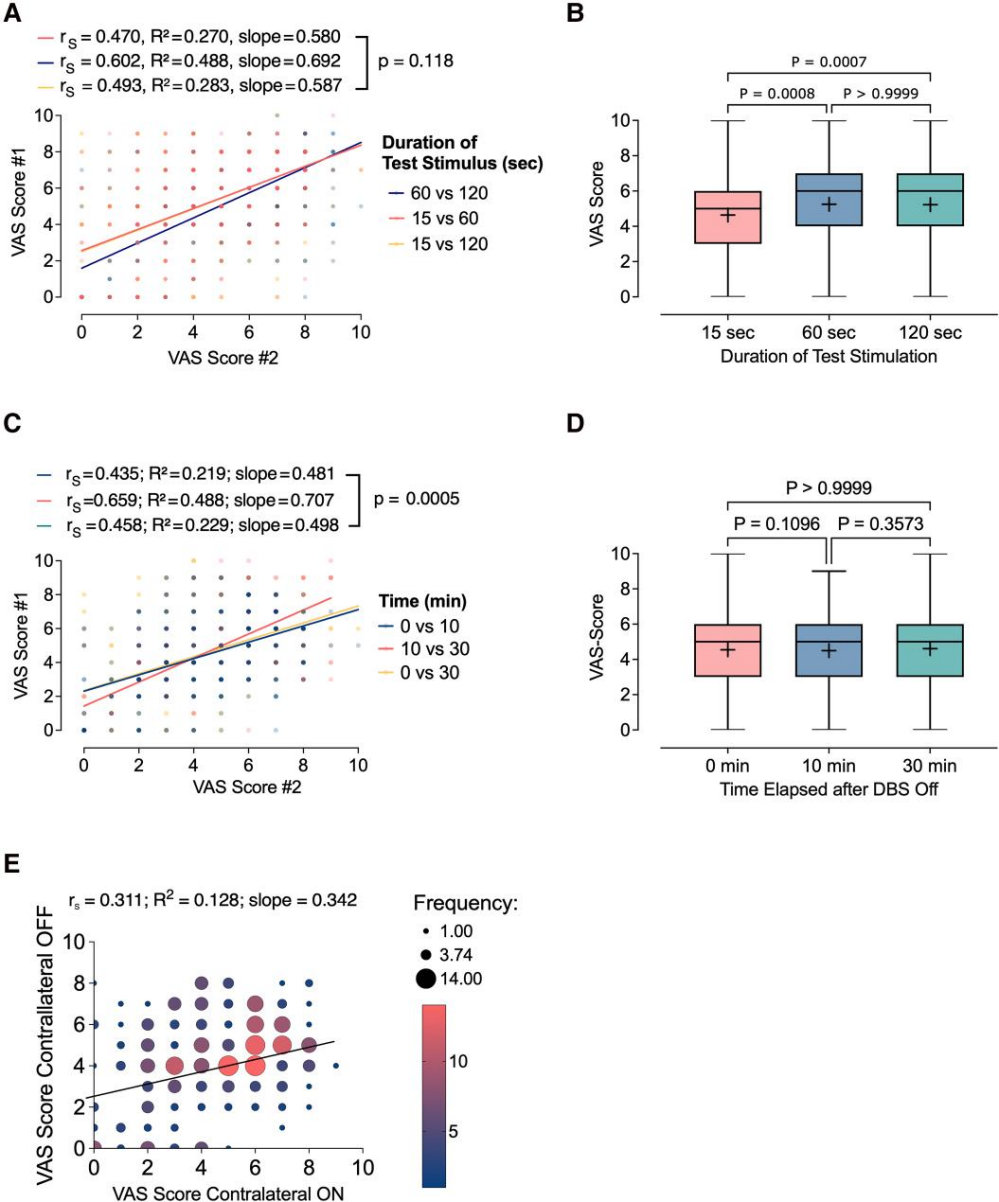
**Effects of test stimulus duration, DBS deactivation interval, and contralateral stimulation status on VAS ratings.** Experimental unit = patient; VAS ratings were aggregated across stimulation programmes per hemisphere. (**A**) Scatter plots showing correlations between VAS scores obtained after different durations of unilateral stimulation (15 s, 60 s, 120 s; n = 382 paired observations per comparison). Linear regression slopes and explained variance (*R*^2^) are annotated. No significant difference in regression slopes was detected (F(21 140) = 2.14, *P* = 0.118), indicating consistent perceptual scaling across durations. Spearman correlations: *r*_S_ = 0.47–0.60, all *P* < 0.0001. (**B**) Boxplots comparing absolute VAS ratings across stimulation durations (15 s, 60 s, 120 s; n = 382 observations per condition). Scores were significantly lower after 15 s compared to 60 s and 120 s (Friedman χ^2^(2) = 15.2, Dunn’s post hoc adjusted *P* < 0.001), whereas 60 s versus 120 s did not differ (*P* > 0.9999). Boxes indicate interquartile range (25th–75th percentile), central line = median, whiskers = 1.5× IQR. (**C**) Scatter plots comparing VAS scores across three DBS-Off intervals (0, 10, 30 min; n = 346 paired observations per comparison). Agreement increased with longer intervals, strongest between 10 and 30 min (*r*_S_ = 0.6590, slope = 0.7078, *R*^2^ = 0.4883). Regression slopes differed significantly (F(21 032) = 7.681, *P* = 0.0005), indicating greater variability at shorter Off periods. (**D**) Group-level comparison of mean VAS scores across DBS-Off intervals (0, 10, 30 min; n = 346 observations per condition). No significant differences were observed (Friedman χ^2^(2) = 6.041, *P* = 0.0488; post hoc *P* > 0.05), consistent with stable subjective ratings despite increased intra-individual variability at shorter Off durations. Boxplots as in (**B**). (**E**) Bubble plot comparing ratings during unilateral stimulation with contralateral side Off versus bilateral stimulation (n = 290 paired observations). Bubble size reflects overlapping repeated observations. Spearman correlation *r*_S_ = 0.3114, *P* < 0.0001; regression slope = 0.3423, *R*^2^ = 0.1282, indicating modest, inter-individually variable influence of contralateral stimulation on subjective perception.

To investigate the impact of stimulation duration on subjective patient feedback, unilateral test stimulation was applied for 15, 60, or 120 s before collecting patient-reported VAS scores. Moderate correlations were found between ratings at different time points: 15 s versus 60 s (*r*_S_ = 0.47, slope = 0.58, *R*^2^ = 0.27), 15 s versus 120 s (r = 0.49, slope = 0.59, *R*^2^ = 0.28), and 60 s versus 120 s (r = 0.60, slope = 0.69, *R*^2^ = 0.49), all *P* < 0.0001 (Fig. 4A). The strongest correlation was observed between 60 and 120 s; however, comparison of regression slopes across all pairs showed no significant difference (F(2, 1140) = 2.14, *P* = 0.118), indicating consistent scaling of subjective ratings regardless of stimulation duration. Despite this, absolute VAS scores differed significantly depending on stimulation length. Scores after 15 s were significantly lower than those after both 60 s (*P* = 0.0008) and 120 s (unpaired median difference 15 versus 60 and 15 versus 120 s. 1.0 [95.0%CI 1.0, 1.0], *P* = 0.0007) whereas no difference was detected between 60 and 120 s (unpaired median difference 0.0 [95.0%CI 0.0, 0.0]; adjusted *P* > 0.9999; Fig. 4B). These results suggest that while the relative pattern of ratings remains stable across durations, shorter stimulation times may systematically underestimate the perceived effect.

### Persistence of Subjective Effects Following DBS Deactivation

To examine whether the duration of DBS deactivation prior to testing influences perceptual ratings, VAS scores were collected following unilateral test stimulation following three different DBS-OFF intervals: immediate testing (0 min), and after delays of 10 and 30 min. Correlational analyses revealed moderate agreement between time points: 0 min versus 10 min (r_S_ = 0.4355, slope = 0.4812, *R*^2^ = 0.2194), 0 min versus 30 min (*r = *0.4587, slope = 0.4988, *R*^2^ = 0.2298), and 10 min versus 30 min (*r = *0.6590, slope = 0.7078, *R*^2^ = 0.4883), all *P* < 0.0001 (Fig. 4C). The strongest correlation and steepest regression slope were found between 10 and 30 min. A formal comparison of regression slopes confirmed significant differences between the conditions (F(2, 1032) = 7.681, *P* = 0.0005), indicating that the consistency of patient ratings varied depending on the DBS-Off interval. Despite these differences in trial-level agreement, the average VAS score remained stable across delays. Group-level VAS scores showed no significant differences between 0 and 10 min (*P* = 0.1096), 0 and 30 min (*P* = 0.3573), or 10 and 30 min (*P* > 0.9999; Fig. 4D) (unpaired median difference for all comparisons 0.0 [95.0%CI 0.0, 0.0]. This was supported by a Friedman test (χ^2^(2) = 6.041, *P* = 0.0488), which did not reveal robust evidence for systematic shifts in mean ratings across time points.

### Effect of Contralateral Stimulation Status

To assess the influence of unilateral versus bilateral stimulation on subjective perception, VAS ratings obtained during unilateral test stimulation with the contralateral side turned OFF were compared to those collected under bilateral stimulation. Each participant completed 36 stimulation trials (18 per hemisphere) at two amplitudes (1 mA and 3 mA), with the contralateral electrode deactivated throughout the contralateral-OFF condition. A statistically significant but modest correlation was found between ratings across the two conditions (r_S_ = 0.3114, *P* < 0.0001), accompanied by a shallow regression slope (0.3423) and low explained variance (*R*^2^ = 0.1282), indicating substantial inter-trial variability (Fig. 4E). While the effect size was small (F(1, 288) = 42.36, *P* < 0.0001), the modest strength of association suggests that the presence or absence of contralateral stimulation alters the subjective experience in a manner that varies across individuals.

## Discussion

Across four complementary experimental paradigms, we demonstrate that VAS ratings exhibit substantial robustness across various experimental conditions. The test–retest analysis indicated moderate within-subject consistency (*r*_S_ = 0.70; *R*^2^ of 0.53) for repeated ratings of identical stimulation settings. This finding challenges the notion that subjective reports in the DBS context are inherently unreliable. Despite the subjective nature of VAS assessments, they yielded systematic and reproducible responses under structured conditions. This is of relevance to clinical programming, where subjective impressions often guide therapeutic decisions, and strongly supports the structured incorporation of patient feedback into routine programming protocols.

Subjective patient feedback may hold value in the setting of remote programming, where direct clinical examination is not applicable. Telemedicine is increasingly employed to deliver care across neurological disorders, including movement disorders.^14-18^ The feasibility of remote DBS programming has been demonstrated in several retrospective^19-25^ and prospective studies,^26^ and randomized controlled studies are underway (NCT05193825). We propose that patient-reported outcome measures, such as VAS-based feedback, should be incorporated into remote care models, and our findings support the reliability of such measures in contexts where conventionally employed clinical signs are not readily available.

A further development in DBS therapy is the emergence of adaptive closed-loop systems guided by electrophysiological feedback.^27-29^ Most approaches rely on beta-band local field potentials (LFPs), which are thought to correlate with bradykinesia in PD.^30^ However, meta-analyses indicate that beta amplitude accounts for only 17% of symptom variability,^31^ raising concerns about its suitability as a sole control signal. We suggest that future studies should investigate the relationship between patient-reported outcomes, including VAS-based feedback, and electrophysiological markers, and assess whether such feedback could complement LFPs to enhance the robustness and clinical utility of closed-loop algorithms. Moreover, our results are relevant for the development and optimization of control algorithms, where temporal parameters—such as the speed at which the algorithm responds to feedback—may substantially influence patient perception.

A noteworthy observation concerns the dissociation between symptom type and VAS reliability. While DBS programming is often guided by motor symptoms that respond quickly and consistently to stimulation,^3,4,11^ tremor-dominant phenotypes—despite their typically rapid and observable improvement—did not predict greater rating consistency (Fig. 3A and B). This was surprising and suggests that VAS ratings may reflect broader perceptual changes beyond the resolution of obvious motor symptoms such as tremor. This suggests that VAS feedback may capture dimensions of DBS effect not fully accounted for by standard motor assessments and could serve as an independent readout of therapeutic efficacy in addition to clinical signs. Tremor-positive subgroups showed lower test–retest reliability of VAS ratings than tremor-negative subgroups, contrary to what might be expected. Although a difference in correlation coefficients was observed (e.g. *r*_S_ = 0.6572 versus *r*_S_ = 0.7233, both *P* < 0.0001), the absolute difference was modest, and the size of the subgroups differed (n = 11 versus 4). Even where statistically significant, it remains unclear whether such differences are large enough to have meaningful clinical implications.

Our findings indicate that the temporal dynamics of DBS testing influence the reliability of patient-reported VAS ratings. Ratings obtained after only 15 seconds of stimulation showed moderate agreement with those acquired at longer durations, whereas the strongest concordance was observed between 60 and 120 seconds (Fig. 4A and B). Conversely, absolute group-level VAS scores were lower at the 15-second interval compared with the 60- and 120-second intervals, indicating that while the relative pattern of ratings remains stable across durations, shorter stimulation times may systematically underestimate perceived effects (Fig. 4A and B). This likely reflects the transient nature of many DBS-induced adverse sensations, which typically resolve within a few seconds.^3,32,33^ Our findings show for the first time that subjective perception stabilizes after approximately 60 s of continuous stimulation. Collectively, these results highlight the importance of allowing at least 60 s of stimulation before collecting patient feedback to ensure reliable assessments. Clinically, this has direct implications for standardizing DBS programming protocols, including structured monopolar reviews, and for validating subjective measures, as premature evaluation may underestimate the full perceptual effects of stimulation.

The influence of DBS washout intervals before testing was also examined. While group-level average VAS scores remained similar across time points (Fig. 4D), regression slopes differed significantly between the 0 versus 10 min, 0 versus 30 min, and 10 versus 30 min intervals (Fig. 4C), indicating that longer DBS-off periods before testing improve the reliability of patient-reported feedback. Clinically, these findings suggest that programming sessions, including monopolar reviews, should commence only after a washout period of at least 10 minutes on the hemisphere being tested.

Contralateral stimulation emerged as a notable confounder (Fig. 4E). Although unilateral and bilateral conditions were statistically correlated, inter-trial variability was substantial, indicating that contralateral stimulation exerts a complex, non-additive influence on subjective perception. Practically, this suggests that turning off the opposite electrode during testing may distort patient feedback; instead, maintaining contralateral stimulation is preferable for reliable evaluations. The modest correspondence observed between contralateral stimulation On versus Off underscores the role of hemispheric interactions in shaping perception. This finding has direct implications for aDBS, where most approaches assume unilateral LFP activity provides an adequate control signal. Our results suggest that unilateral signals may not fully capture the bilateral network dynamics underlying motor and perceptual states, and that optimization of aDBS may require cross-hemispheric integration, or network-level biomarkers rather than reliance on a single hemisphere. However, the observed correlation between on and off VAS ratings was modest in magnitude, indicating a measurable but potentially limited clinical impact.

Crucially, neither disease severity nor cognitive performance appeared to affect the reliability of VAS ratings (Fig. 3C; Supplementary Fig. 1). Even in individuals with greater motor disability or mild cognitive impairment, subjective reports remained consistent, supporting the applicability of VAS across the clinical spectrum of PD. This suggests that structured patient-reported outcomes may retain their utility even in populations often considered less suitable for self-report paradigms. While point estimates were numerically higher in cognitively unimpaired participants, these differences did not reach statistical significance. Thus, within the limits of the present sample, cognitive status was not associated with detectable differences in VAS test–retest reliability. Given the limited sample size, these findings may be interpreted as an absence of detectable evidence rather than as conclusive evidence that cognitive impairment does not affect retest reliability.

### Limitations

Several limitations should be noted. First, the study was conducted with a relatively small and homogeneous cohort (*N* = 15), which may limit generalizability. Despite the small sample size, the high number of repeated VAS ratings (>3000) enabled robust within-subject reliability analyses. Future studies should aim to replicate these findings in larger, multicentre cohorts, including more diverse PD populations with varying symptom profiles and cognitive abilities.

Second, while the study was performed under highly controlled experimental conditions, real-world DBS programming often involves additional confounds such as fatigue, medication fluctuations, and time constraints, which may affect the stability of patient-reported feedback.

Third, the subgroup analyses were conducted post hoc and are exploratory in nature. The same small cohort of participants was stratified multiple times across different subgroup definitions, so the subgroups are not statistically independent. This increases the risk of false positives, and the findings should be interpreted with caution. Moreover, although many VAS ratings were collected per participant, the number of independent participants was low. Therefore, statistical significance driven by repeated within-subject testing should not be overinterpreted as evidence of clinical relevance. While these analyses provide preliminary insights into factors influencing VAS reliability, such as motor phenotype or tremor severity, they are not confirmatory. Future studies with prespecified hypotheses and larger cohorts are needed to validate these observations.

A further limitation concerns participant dropout across experimental paradigms. While fifteen participants completed the primary reliability experiment, fewer participants completed subsequent experiments, particularly the contralateral stimulation condition. This reduction in sample size may have limited statistical power for some secondary analyses and may reduce the generalizability of findings related to stimulation duration, DBS washout effects, and contralateral stimulation. Future studies should aim to minimize participant burden and employ multicentre designs to ensure more complete datasets across experimental conditions.

## Conclusions

Visual analogue scales provide reliable and quantifiable patient feedback during STN-DBS programming, with reliability influenced by motor phenotype, stimulation duration, and bilateral context. These findings support the integration of structured VAS feedback into routine programming and emerging closed-loop DBS systems. Future work should focus on larger, multicentre studies to validate these results, explore their generalizability to broader PD populations, and assess the utility of VAS-based feedback in remote and adaptive DBS programming workflows.

## Supplementary Material

fcag100_Supplementary_Data

## Data Availability

The data that support the findings of this study are available from the corresponding author, upon reasonable request.
