# Supplementary material for: Psychometric reliability of patient-reported visual analogue scales in subthalamic nucleus deep brain stimulation programming for Parkinson’s disease
Source: Brain Commun. 2026 Mar 19;8(2):fcag100. doi: 10.1093/braincomms/fcag100 (PMC13037577; doi:10.1093/braincomms/fcag100)
Supplement: fcag100_Supplementary_Data [file fcag100_supplementary_data.pdf]

Supplementary Material:

Psychometric reliability of patient-reported visual analogue scales in STN-DBS programming for Parkinson's disease

Off et al.

Supplemental Figure 1

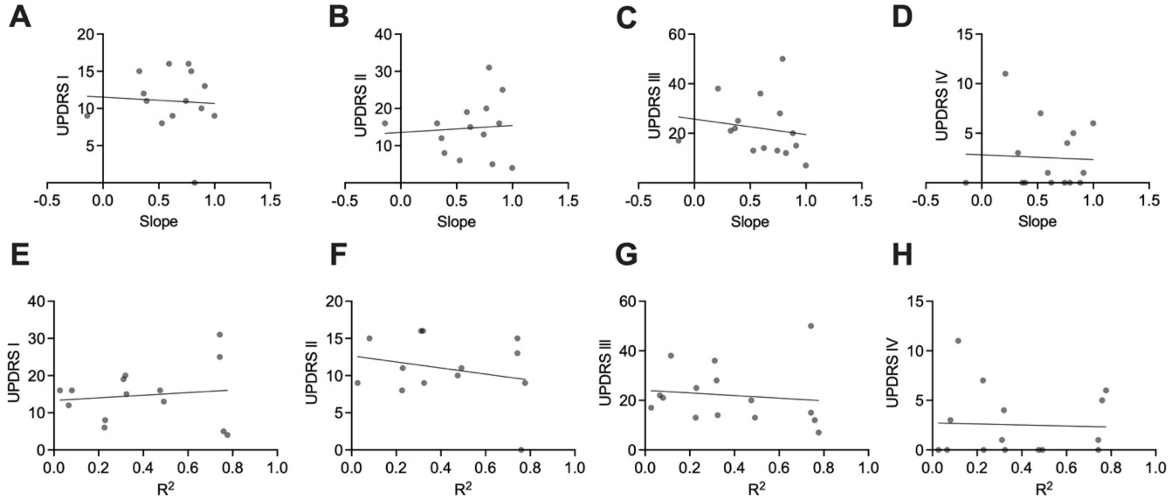

Supplementary Fig. 1. No significant relationship between disease severity and reliability of subjective ratings. Experimental unit = patient; VAS slopes were calculated per patient across stimulation programs.

(**A–D**) Scatter plots showing Spearman correlations between test–retest reliability (slope of VAS scores) and UPDRS Part I (A), Part II (B), Part III (C), and Part IV (D). Correlation coefficients were low and non-significant:

Part I: $r_s = –0.06$, $p = 0.84$

Part II: $r_s = 0.10$, $p = 0.74$

Part III: $r_s = –0.20$, $p = 0.48$

Part IV: $r_s = –0.05$, $p = 0.87$

(**E–H**) Linear regression plots assessing the predictive relationship between each UPDRS subscore and VAS test–retest slopes. No model showed a significant association; slopes (95% CI), R^2, and F-statistics:

Part I: slope = –0.85 [–9.55, 7.85], $R^2 = 0.0038$, $F(1,13) = 0.045$, $p = 0.835$

Part II: slope = 3.53 [–14.13, 21.19], $R^2 = 0.0156$, $F(1,13) = 0.18$, $p = 0.671$

Part III: slope = –5.31 [–30.96, 20.34], $R^2 = 0.0152$, $F(1,13) = 0.19$, $p = 0.662$

Part IV: slope = –0.51 [–8.02, 7.00], $R^2 = 0.0017$, $F(1,13) = 0.02$, $p = 0.885$

Shaded areas represent 95% confidence intervals. Each point represents one patient-level slope derived from repeated VAS ratings.

Supplemental Table 1:

Experiment I: Test

Abbott:

Left Electrode		
**Contact**	**Amplitude**	**VAS**
3B	3	
2A	3	
3B	1	
2B	1	
3C	1	
1	3	
3C	3	
3A	1	
4	3	
2B	3	
2A	1	
OFF	OFF	
2C	1	
2C	3	
1	1	
3A	3	
4	1	
OFF	OFF	

Right Electrode		
**Contact**	**Amplitude**	**VAS**
11A	3	
11B	3	
10C	3	
10A	3	
10C	1	
10A	1	
11B	1	
10B	3	
11A	1	
11C	3	
OFF	OFF	
9	1	
11C	1	
OFF	OFF	
12	3	
10B	1	
9	3	
12	1	

Boston Scientific:

Left Electrode		
**Contact**	**Amplitude**	**VAS**
1	3	
5	3	
5	1	
2	3	
OFF	OFF	
8	3	
8	1	
6	1	
6	3	
2	1	
3	3	
1	1	
7	3	
4	3	

Right Electrode		
**Contact**	**Amplitude**	**VAS**
3	3	
OFF	OFF	
5	3	
3	1	
1	1	
7	3	
1	3	
8	3	
5	1	
2	3	
4	3	
7	1	
2	1	
8	1	

4	1	
3	1	
7	1	
OFF	OFF	

OFF	OFF	
6	3	
4	1	
6	1	

Medtronic:

Left Electrode		
**Contact**	**Amplitude**	**VAS**
1	3	
0	1	
OFF	OFF	
3	1	
OFF	OFF	
0	3	
2	3	
1	1	
3	3	
2	1	

Right Electrode		
**Contact**	**Amplitude**	**VAS**
11	3	
OFF	OFF	
10	1	
10	3	
9	1	
9	3	
11	1	
8	1	
8	3	
OFF	OFF	

Medtronic Directional:

Left Electrode		
**Contact**	**Amplitude**	**VAS**
1A	1	
1C	3	
2C	1	
2B	3	
1A	3	
1C	1	
0	3	
2C	3	
3	3	
1B	1	
2A	3	
2A	1	
3	1	
1B	3	
OFF	OFF	
0	1	
OFF	OFF	
2B	1	

Right Electrode		
**Contact**	**Amplitude**	**VAS**
OFF	OFF	
10C	3	
OFF	OFF	
10A	1	
9A	3	
10B	3	
9B	1	
8	1	
8	3	
11	1	
10A	3	
10C	1	
9C	3	
10B	1	
9B	3	
9A	1	
9C	1	
11	3	

<u>Experiment I: Retest</u>

Abbott

Left Electrode		
**Contact**	**Amplitude**	**VAS**
4	1	
2A	3	
2B	3	
4	3	
3B	3	
AUS	AUS	
3C	3	
3A	3	
2C	1	
3C	1	
2A	1	
3B	1	
1	1	
2C	3	
2B	1	
1	3	
AUS	AUS	
3A	1	

Right Electrode		
10A	1	
10A	3	
AUS	AUS	
11A	3	
11C	3	
11B	1	
10C	3	
12	1	
AUS	AUS	
10B	3	
11C	1	
9	1	
10B	1	
12	3	
10C	1	
9	3	
11B	3	
11A	1	
10A	1	

Boston:

Left Electrode		
**Contact**	**Amplitude**	**VAS**
2	1	
1	3	
1	1	
7	3	
OFF	OFF	
6	1	
4	1	
2	3	
6	3	
8	3	
8	1	
5	3	
3	1	
5	1	
3	3	
4	3	
7	1	

Right Electrode		
**Contact**	**Amplitude**	**VAS**
2	3	
8	1	
OFF	OFF	
2	1	
4	1	
8	3	
4	3	
3	3	
6	3	
3	1	
7	1	
5	1	
7	3	
1	3	
5	3	
OFF	OFF	
6	1	

OFF	OFF		1	1	

Medtronic

Left Electrode		
**Contact**	**Amplitude**	**VAS**
0	3	
OFF	OFF	
1	1	
3	3	
3	1	
OFF	OFF	
2	3	
2	1	
1	3	
0	1	

Right Electrode		
**Contact**	**Amplitude**	**VAS**
9	3	
8	1	
OFF	OFF	
9	1	
11	3	
10	1	
11	1	
OFF	OFF	
8	3	
10	3	

Medtronic Directional:

Left Electrode		
**Contact**	**Amplitude**	**VAS**
1C	3	
1C	1	
2B	1	
2B	3	
OFF	OFF	
0	3	
3	1	
2A	1	
1A	3	
OFF	OFF	
2C	3	
1B	3	
1B	1	
1A	1	
2C	1	
3	3	
2A	3	
0	1	

Right Electrode		
**Contact**	**Amplitude**	**VAS**
10B	1	
8	3	
11	3	
9C	1	
10C	1	
9B	1	
OFF	OFF	
10C	3	
8	1	
9C	3	
9A	3	
10B	3	
11	1	
9A	1	
10A	1	
10A	3	
9B	3	
OFF	OFF	

Supplemental Table 2:

Experiment II: Test Stimulation for t_{Test} = 60 sec

Abbott:

Left Electrode		
**Contact**	**Amplitude**	**VAS**
3B	1	
2B	1	
3A	3	
1	3	
2A	1	
2B	3	
3C	1	
OFF	OFF	
OFF	OFF	
3C	3	
2C	1	
4	3	
4	1	
1	1	
2A	3	
2C	3	
3B	3	
3A	1	

Right Electrode		
**Contact**	**Amplitude**	**VAS**
11A	3	
12	3	
10A	1	
10A	3	
9	1	
11A	1	
11B	3	
10B	1	
10C	1	
OFF	OFF	
10B	3	
12	1	
11C	1	
9	3	
11C	3	
10C	3	
11B	1	
OFF	OFF	

Boston:

Left Electrode		
**Contact**	**Amplitude**	**VAS**
8	3	
4	3	
8	1	
3	1	
1	1	
2	1	
7	3	
5	1	
7	1	
OFF	OFF	
OFF	OFF	
5	3	
1	3	

Right Electrode		
**Contact**	**Amplitude**	**VAS**
OFF	OFF	
8	3	
1	1	
1	3	
OFF	OFF	
2	1	
6	1	
7	1	
4	1	
5	3	
5	1	
3	1	
8	1	

6	1		7	3	
4	1		3	3	
3	3		2	3	
6	3		4	3	
2	3		6	3	

Medtronic:

Left Electrode		
**Contact**	**Amplitude**	**VAS**
3	1	
3	3	
2	3	
2	1	
OFF	OFF	
1	1	
0	3	
OFF	OFF	
0	1	
1	3	

Right Electrode		
**Contact**	**Amplitude**	**VAS**
10	3	
10	1	
8	3	
11	1	
8	1	
9	3	
9	1	
OFF	OFF	
11	3	
OFF	OFF	

Medtronic Directional:

Left Electrode		
**Contact**	**Amplitude**	**VAS**
2B	1	
2C	1	
1A	3	
3	3	
1B	1	
OFF	OFF	
1C	1	
1B	3	
2A	3	
0	3	
2C	3	
3	1	
OFF	OFF	
0	1	
2B	3	
1C	3	
2A	1	
1A	1	

Right Electrode		
**Contact**	**Amplitude**	**VAS**
9B	1	
10A	3	
11	1	
11	3	
9A	3	
8	3	
9B	3	
10C	3	
9C	3	
9C	1	
OFF	OFF	
9A	1	
10B	3	
8	1	
OFF	OFF	
10C	1	
10A	1	
10B	1	

Experiment II: Test Stimulation for t_{Test} = 120 sec

Abbott:

Left Electrode		
**Contact**	**Amplitude**	**VAS**
2A	3	
4	1	
1	3	
4	3	
2B	1	
3C	3	
2A	1	
2C	3	
3B	3	
3C	1	
OFF	OFF	
3A	3	
3A	1	
1	1	
OFF	OFF	
3B	1	
2C	1	
2B	3	

Right Electrode		
**Contact**	**Amplitude**	**VAS**
10B	3	
OFF	OFF	
11B	3	
9	3	
10C	3	
11C	1	
11B	1	
12	3	
11A	3	
11C	3	
10A	1	
12	1	
10A	3	
11A	1	
10B	1	
9	1	
10C	1	
OFF	OFF	

Boston:

Left Electrode		
**Contact**	**Amplitude**	**VAS**
OFF	OFF	
7	1	
OFF	OFF	
6	1	
1	1	
4	1	
1	3	
2	3	
7	3	
4	3	
8	3	
3	1	
2	1	
3	3	
6	3	

Right Electrode		
**Contact**	**Amplitude**	**VAS**
5	3	
OFF	OFF	
3	3	
4	1	
6	3	
8	1	
1	1	
7	3	
5	1	
OFF	OFF	
2	3	
7	1	
2	1	
3	1	
1	3	

5	1	
5	3	
8	1	

4	3	
6	1	
8	3	

Medtronic:

Left Electrode		
**Contact**	**Amplitude**	**VAS**
1	1	
2	3	
OFF	OFF	
1	3	
OFF	OFF	
3	3	
3	1	
0	3	
2	1	
0	1	

Right Electrode		
**Contact**	**Amplitude**	**VAS**
10	3	
10	1	
OFF	OFF	
8	3	
9	1	
11	1	
9	3	
OFF	OFF	
11	3	
8	1	

Medtronic Directional:

Left Electrode		
**Contact**	**Amplitude**	**VAS**
1B	1	
0	3	
2A	3	
3	3	
2A	1	
2B	3	
1C	1	
0	1	
2B	1	
OFF	OFF	
1C	3	
1A	3	
3	1	
1B	3	
1A	1	
2C	1	
2C	3	
OFF	OFF	

Right Electrode		
**Contact**	**Amplitude**	**VAS**
10B	3	
10A	3	
10B	1	
9C	1	
OFF	OFF	
10A	1	
9B	1	
11	3	
9C	3	
9A	1	
9A	3	
9B	3	
8	3	
OFF	OFF	
8	1	
11	1	
10C	3	
10C	1	

Supplemental Table 3:

<u>Experiment III: Effect of pausing stimulation prior to VAS testing – t_{OFF} = 10 min</u>

Abbott:

Left Electrode		
**Contact**	**Amplitude**	**VAS**
3C	1	
2B	1	
2B	3	
3A	3	
4	3	
2C	1	
OFF	OFF	
1	1	
2C	3	
3C	3	
3B	1	
4	1	
OFF	OFF	
3A	1	
2A	3	
1	3	
3B	3	
2A	1	

Right Electrode		
**Contact**	**Amplitude**	**VAS**
9	3	
10A	1	
12	3	
11B	3	
OFF	OFF	
9	1	
12	1	
11A	1	
10C	1	
11C	3	
10B	3	
10C	3	
OFF	OFF	
11C	1	
10A	3	
11A	3	
11B	1	
10B	1	

Boston:

Left Electrode		
**Contact**	**Amplitude**	**VAS**
OFF	OFF	
8	1	
2	1	
6	1	
5	3	
2	3	
3	1	
5	1	
OFF	OFF	
3	3	
7	3	

Right Electrode		
**Contact**	**Amplitude**	**VAS**
3	1	
6	3	
1	3	
OFF	OFF	
2	3	
1	1	
OFF	OFF	
7	1	
8	1	
2	1	
5	1	

6	3	
8	3	
7	1	
4	1	
1	3	
4	3	
1	1	

7	3	
4	1	
6	1	
4	3	
5	3	
3	3	
8	3	

Medtronic:

Left Electrode		
**Contact**	**Amplitude**	**VAS**
2	1	
1	1	
0	3	
3	1	
2	3	
1	3	
OFF	OFF	
OFF	OFF	
3	3	
0	1	

Right Electrode		
**Contact**	**Amplitude**	**VAS**
8	1	
10	1	
8	3	
9	3	
OFF	OFF	
10	3	
OFF	OFF	
11	3	
9	1	
11	1	

Medtronic Segmente:

Left Electrode		
**Contact**	**Amplitude**	**VAS**
1A	1	
0	3	
1B	3	
1C	3	
0	1	
2B	3	
2C	3	
3	1	
2B	1	
2C	1	
OFF	OFF	
1A	3	
2A	3	
1C	1	
1B	1	
2A	1	
OFF	OFF	

Right Electrode		
**Contact**	**Amplitude**	**VAS**
10C	1	
8	1	
10B	1	
OFF	OFF	
8	3	
9B	1	
10A	1	
9B	3	
9A	1	
9C	1	
11	1	
10A	3	
9C	3	
10B	3	
9A	3	
OFF	OFF	
10C	3	

3	3			11	3	

Experiment III: Effect of pausing stimulation prior to VAS testing – t_{OFF} = 30 min

Abbott:

Left Electrode		
**Contact**	**Amplitude**	**VAS**
4	3	
1	1	
2A	1	
3B	1	
3A	3	
2A	3	
3B	3	
2C	3	
3C	3	
2B	3	
2B	1	
OFF	OFF	
1	3	
OFF	OFF	
3C	1	
4	1	
3A	1	
2C	1	

Right Electrode		
**Contact**	**Amplitude**	**VAS**
10B	3	
9	1	
11C	1	
10A	1	
10C	1	
OFF	OFF	
12	3	
11B	3	
11C	3	
11B	1	
11A	3	
10C	3	
10B	1	
12	1	
11A	1	
10A	3	
OFF	OFF	
9	3	

Boston:

Left Electrode		
**Contact**	**Amplitude**	**VAS**
6	3	
8	1	
6	1	
3	1	
1	1	
1	3	
4	3	
7	3	
7	1	
2	3	
5	3	

Rechts		
**Contact**	**Amplitude**	**VAS**
1	1	
7	1	
3	3	
OFF	OFF	
6	1	
5	3	
7	3	
5	1	
6	3	
8	3	
3	1	

OFF	OFF		2	1	
4	1		4	3	
2	1		1	3	
5	1		4	1	
8	3		8	1	
3	3		OFF	OFF	
OFF	OFF		2	3	

Medtronic:

Left Electrode		
**Contact**	**Amplitude**	**VAS**
1	1	
3	3	
0	1	
OFF	OFF	
0	3	
2	1	
OFF	OFF	
1	3	
3	1	
2	3	

Right Electrode		
**Contact**	**Amplitude**	**VAS**
OFF	OFF	
8	1	
10	1	
11	1	
9	3	
10	3	
8	3	
OFF	OFF	
11	3	
9	1	

Medtronic Segmente:

Left Electrode		
**Contact**	**Amplitude**	**VAS**
1C	1	
0	3	
1A	1	
1A	3	
2B	3	
2A	1	
1C	3	
2C	1	
3	1	
1B	1	
1B	3	
2C	3	
OFF	OFF	
2B	1	
2A	3	

Right Electrode		
**Contact**	**Amplitude**	**VAS**
10C	1	
11	1	
OFF	OFF	
9C	1	
10A	3	
8	1	
10B	3	
9B	1	
9B	3	
8	3	
10C	3	
OFF	OFF	
10B	1	
9C	3	
9A	1	

3	3			10A	1	
0	1			9A	3	
OFF	OFF			11	3	

Supplemental Table 4:

<u>Experiment IV: Contralateral Stim OFF</u>

Abbott:

**Left Electrode**		
**Contact**	**Amplitude**	**VAS**
2B	1	
3A	1	
2C	3	
OFF	OFF	
2A	3	
3C	3	
2A	1	
3A	3	
OFF	OFF	
3B	3	
4	3	
1	3	
2B	3	
4	1	
3C	1	
1	1	
3B	1	
2C	1	

**Right Electrode**		
**Contact**	**Amplitude**	**VAS**
10B	3	
11A	1	
11C	1	
11C	3	
10C	3	
9	1	
10A	1	
11B	1	
10B	1	
OFF	OFF	
11B	3	
10A	3	
11A	3	
9	3	
12	3	
12	1	
10C	1	
OFF	OFF	

Boston:

**Left Electrode**		
**Contact**	**Amplitude**	**VAS**
3	3	
4	1	
3	1	
1	1	
OFF	OFF	
1	3	
5	3	

**Right Electrode**		
**Contact**	**Amplitude**	**VAS**
1	3	
4	3	
7	3	
3	3	
8	3	
OFF	OFF	
4	1	

7	1	
4	3	
5	1	
2	1	
7	3	
2	3	
8	3	
8	1	
6	3	
6	1	
OFF	OFF	

8	1	
6	3	
OFF	OFF	
3	1	
5	1	
5	3	
6	1	
2	3	
1	1	
7	1	
2	1	

Medtronic:

Left Electrode		
**Contact**	**Amplitude**	**VAS**
2	1	
0	1	
OFF	OFF	
1	1	
0	3	
2	3	
3	1	
3	3	
OFF	OFF	
1	3	

Right Electrode		
**Contact**	**Amplitude**	**VAS**
11	1	
8	3	
10	3	
11	3	
OFF	OFF	
OFF	OFF	
9	3	
9	1	
10	1	
8	1	

Medtronic Segmente:

Left Electrode		
**Contact**	**Amplitude**	**VAS**
1B	1	
2B	3	
0	3	
1C	1	
3	1	
1C	3	
OFF	OFF	
2A	3	
1A	3	
2C	3	
1B	3	
2C	1	
1A	1	

Right Electrode		
**Contact**	**Amplitude**	**VAS**
OFF	OFF	
9A	3	
10A	3	
8	1	
OFF	OFF	
10B	1	
8	3	
9B	3	
10C	3	
10A	1	
9C	1	
10C	1	
11	1	

0	1	
3	3	
2B	1	
2A	1	
OFF	OFF	

11	3	
9A	1	
10B	3	
9C	3	
9B	1	

Supplemental Table 1-4 Tables illustrating the experimental protocol spread sheets used to sample VAS ratings in the different experiments I-IV.
